# Supplementary material for: Are carbon emissions trading and green financial instruments synergistic? -Comprehensive quantitative research based on content analysis
Source: PLoS One. 2024 Mar 7;19(3):e0298601. doi: 10.1371/journal.pone.0298601 (PMC10919601; doi:10.1371/journal.pone.0298601)
Supplement: S1 Data — (ZIP) [file pone.0298601.s001.zip › Data/地级市Z_F工作报告.zip!/╔╜╢1⁄2/╟α╡║╩╨/└┤╫╘╣1⁄2╓┌║┼í╛┬φ┐╦╩2╛▌═°í┐_2008.docx]

各位代表：

　　现在,我代表青岛市第十三届人民政府向大会作工作报告,请予审议，并请各位政协委员和其他列席人员提出意见。

　　一、五年的工作回顾

　　过去的五年，是青岛发展很不平凡的五年。在中共青岛市委的正确领导下，在市人大、市政协的监督支持下，我们紧紧依靠全市人民，坚持以邓小平理论和“三个代表”重要思想为指导，全面贯彻科学发展观，认真落实宏观调控政策，以人为本，执政为民，恪尽职守，不辱使命，圆满完成市十三届人大历次会议确定的各项任务。五年来，我们集中财力物力，办成了一些多年想办而没有条件办的民生实事，解决了一些多年制约经济社会发展的历史难题，制度建设和体制创新迈出重要步伐，为全面建设小康社会打下了坚实基础。

　　过去的五年，是综合实力显著增强、经济发展向又好又快积极转变的五年。初步预计，全市生产总值年均增长16%以上，地方财政一般预算收入年均增长28.9%，总量分别是2002年的2.1倍和2.9倍。人均生产总值达到4.5万元，是2002年的2.2倍。二氧化硫、化学需氧量排放量持续下降，节能减排成效显现。

　　过去的五年，是民生质量持续改善、人民生活向全面小康加快迈进的五年。城市居民人均可支配收入和农民人均纯收入年均增长15.4%、12.2%，是2002年的2倍、1.8倍。惠及全民的就业、医疗、教育、住房等民生保障体系初步形成。公共安全应急体系基本建立。全部取消农业税。实现了城乡免费义务教育。建立了被征地农民养老保险制度。城镇居民基本医疗保险实现市区全覆盖，新型农村合作医疗参合率达到98%。率先建立面向城市低收入家庭的住房保障制度。

　　过去的五年，是城乡面貌明显变化、城市功能向现代化快速提升的五年。以筹办奥帆赛为契机，加快城乡基础设施建设，城市化水平提高5.9个百分点。青岛港跻身世界十大港口之列，市域“一小时经济圈”基本形成。青岛奥林匹克帆船中心、市立医院东院区、山东国际农展交易中心、高级技工学校等建成使用；市体育运动学校、老年大学、图书馆、群众艺术馆、博物馆、美术馆、青岛音乐厅等改扩建完成；青岛大剧院、市体育馆、游泳跳水馆等开工建设。率先实现有线数字电视整体转换。

　　过去的五年，是改革开放逐步深化、体制机制向市场经济加速转轨的五年。以改善民生为重点的公共财政体系开始建立。搭建了国企职工安置平台。国有资产监管体制基本形成。国有中小企业改制面达到96%。民营经济占全市生产总值比重提高9.7个百分点。全方位对外开放格局进一步完善。外贸进出口总额、实际到账外资和内资分别是2002年的3.1倍、3.8倍和3.2倍。新增两个国家级出口加工区。与世界21个城市结为友好城市或友好合作关系城市。成功承办亚洲合作对话第三次外长会议、第二届中日韩旅游部长会议等重要国际会议。

　　回顾五年，主要做了以下工作：

　　（一）推动产业结构调整，确保经济平稳较快发展

　　五年来，以稳定优化一产、调整提升二产、加快发展三产为着力点，协调推进先进制造业、现代服务业和现代农业。坚持龙头企业发展、大项目带动与产业集群配套同步实施，加快建设符合国家产业政策的重大项目，着力优化投资结构，提高园区集约化水平，家电电子、汽车机车、造修船、海洋工程、石油化工等制造业集群初具规模。坚持重点推进“九大产业”、实现“五个结合”、打造“三个载体”的服务业发展思路，金融、物流、旅游、会展等快速发展。金融规模稳健扩张，引进金融机构36家，2007年末全市存、贷款余额均为2002年的2.4倍。建成极地海洋世界等投资过亿元旅游项目31个，旅游业总收入和接待国内外游客分别是2002年的2.7倍、1.7倍。中国国际消费电子博览会、青岛国际啤酒节的影响力进一步提高。坚持高效、生态、安全，大力发展现代农业，规划建设四个100万亩优质农产品生产基地，建成100万亩粮食示范区。

　　（二）加快发展方式转变，促进速度质量效益相协调

　　五年来，以自主创新、节能减排、环境保护、集约用地和质量品牌为突破口，加快建设创新型城市、资源节约型和环境友好型社会。突出企业主体地位，加快科研院所引进，努力突破关键技术，取得网络家电标准、液晶模组、数字电视芯片、高速动车组等重大创新成果。国家级企业技术中心达到12家。引进6家国家级科研机构。大力发展循环经济，在全国率先完成铬渣安全处置，成功破解钢渣、白泥资源化利用难题，海水源热泵、海水淡化实现突破。生态市建设全面启动，空气质量优良率由88%提高到91.2%。强化土地管理，严格落实经营性和工业用地招拍挂制度。大力实施品牌战略和标准化战略，新增中国世界名牌产品2个、中国名牌产品53个、中国驰名商标17个，9项标准成为国际标准提案。

　　（三）强化规划建设管理，提高城市现代化水平

　　五年来，以科学规划、集约建设、精细管理为抓手，不断提升城市品质，加快城乡一体化进程。城市规划强化科学性、先导性和权威性，市区控制性规划覆盖率达到100%。城市建设强化质量意识、精品意识和保护意识，机场扩建、东西快速路、胶济铁路电气化改造等项目竣工使用。胶州湾环境保护、前海一线截污、浮山综合整治加快实施。市区北部基础设施建设力度加大。加强城市风貌和优秀历史建筑保护。城市管理强化法制化、人性化和属地化，健全“两级政府、三级管理、四级网络”的管理体制，提高了综合管理水平。

　　（四）统筹城乡协调发展，扎实推进新农村建设

　　五年来，以促进农民增收为核心，着力改善农村基础条件，加快公共财政向“三农”倾斜，市级财政用于“三农”投入51亿元，年均增长31%。全面落实各项惠农政策。免除农民负担6.96亿元，发放惠农补贴5.16亿元。镇村小学校舍、医疗卫生和计生服务基础设施标准化建设基本完成。“村村通自来水”、“村村通广播电视”、“镇镇设文化站、图书馆”、“镇镇有敬老院”基本实现。新型农村合作医疗共为农民报销医疗费4.36亿元，被征地农民养老参保率达到96%。实施了库区移民村帮扶工程。以加快新型工业化为主导，大力推进产业项目向县域布局。县域经济总量占全市比重由56%提高到63%。

　　（五）深入推进改革开放，不断增强经济发展活力

　　五年来，以市场化为导向，加快制度创新。对33户市属企业实施重点监管，将市政公用、交通等行业符合条件的国有资产纳入监管范围。国有大企业产权多元化改革稳步推进。统筹国有资产出让收益、企业土地变现收入和财政投入37.3亿元，解决了11.8万职工分流安置和5.2万人次的欠薪问题。调整了市对区市的财政管理体制。农村综合配套改革试点有序推进。完善促进民营经济发展政策。民营企业上市实现“零突破”，上市公司达到6家。建立吸引外资与旅游促销、奥运推介和城市宣传“四位一体”的新机制。引进投资过亿美元项目17个,服务业利用外资比重达到47%。机电产品出口比重提高8.3个百分点。坚持按制度运行、按程序操作、按章程办事、按规范服务，进一步优化开放环境。区港联动试点通过验收运营，口岸开放进一步扩大。在青外国人的居住、教育、就医条件不断改善。友好城市交流广泛开展，对台、侨务工作取得新成效。对日喀则、曹县等地区的对口帮扶力度不断加大。

　　（六）着力解决民生问题，推动社会事业全面进步

　　五年来，以解决群众最关心、最直接、最现实的利益问题为首要任务和重大职责，着力提高民生质量，切实维护社会公平。全市财政投向社会民生总额740亿元，占同期地方财政一般预算收入的78.2%，是民生投入力度最大、城乡居民受益最多的时期之一。

　　努力为群众办实事。在城乡建设和群众生活方面安排实事61件，投入超过45亿元。坚持以创业促就业，有就业能力和愿望的困难群体得到充分安置，城镇登记失业率控制在3%以内。两次调整最低工资标准，三次提高城乡最低生活保障标准，四次提高企业退休人员养老金水平，分别提高48.8%、42.9%、62.4%。完善了城乡救助体系。坚持每年为老年人办实事，实行免费乘公交车、进公园，实施养老服务“双千”计划，建立了80岁以上老人体检补助制度。坚持公交优先，更新置换公交车1200辆，新增和调整线路115条，公交车多年占路停放扰民明显缓解。市区集中供热突破3000万平方米，普及率由22%提高到44%。

　　繁荣发展社会事业。完善农村义务教育经费保障机制，实现了县域内教师同工同酬。调整了市区义务教育管理体制，全面完成义务教育标准化建设工程。建立“工学结合、半工半读”的职业教育模式，职普比例达到51∶49。引进3所高校，全日制普通高校在校学生达到26.8万人,是2002年的2.7倍。建立了覆盖各级各类教育的家庭经济困难学生资助体系。城乡公共卫生网络进一步健全，社区卫生服务中心标准化建设基本完成，医疗费用控制初见成效，居民就医条件得到改善。实施了“食品药品放心工程”。社区全部设立文化活动中心，青岛文化街、达尼画家村等成为全国文化产业示范基地，区市全部进入省文化先进县区行列。实现电视高清直播，提高了广播数字音频转播能力。电视公共节目频道由27套增加到60套。全民健身服务体系日趋完善，建成迎奥体育街，增设1400多条健身路径。人口与计划生育综合改革全面推进，低生育水平保持稳定。促进妇女、儿童、残疾人事业健康发展，积极做好民族宗教工作，司法、地震、档案、史志、仲裁工作进一步加强。

　　（七）加强精神文明建设，努力维护社会和谐稳定

　　五年来，以提高市民文明素质和城市文明程度为目标，深入实践社会主义荣辱观，大力实施人文奥运工程，广泛开展文明村镇、文明社区、文明企业等群众性精神文明创建活动，营造了“同迎奥帆赛，共建文明城”的浓厚氛围。我市进入首批“全国文明城市”行列。未成年人思想道德建设进一步加强。国防动员、人民防空和民兵预备役建设取得新成效，圆满完成中俄军事演习和全国民兵新大纲集训演练保障任务。

　　积极营造和谐稳定的社会环境。坚持安全发展，确保产业项目安全建设、国有资产安全经营、金融信贷安全运行、财政资金安全使用、公共事件安全处置、各类企业安全生产。“平安青岛”建设加快推进，刑事案件、治安案件、集体上访和安全生产事故发生起数整体下降，公众安全感满意率逐年提高，我市被评为“全国社会治安综合治理优秀城市”。强化社区民主管理，不断拓展社区工作新领域。取得抗击非典全面胜利，加快公共安全应急体系建设，在抵御禽流感，抗击“麦莎”、“韦帕”强台风，保障重大国际会议中得到检验和完善。全面加强安监工作，安全生产形势稳定好转。

　　（八）推进政府改革创新，进一步增强行政能力

　　五年来，以建设法治、责任、廉洁、高效政府为目标，坚持依法行政，促进政府工作法制化、程序化、规范化。认真执行人大及其常委会的决议决定，坚持重大事项报告制度，自觉接受法律监督和工作监督。坚持重大问题与人民政协协商制度，自觉接受民主监督。认真办理人大代表议案、建议和政协提案、建议案。及时把人民群众反映的突出问题纳入政府议事日程，进一步健全重大决策专家咨询、社会公示和听证制度，完善了科学民主决策机制。提请市人大常委会审议通过地方性法规36件，制定政府规章41件。健全政府新闻发布制度，重大民生项目办理情况向社会公布。开办《行风在线》节目，通过“我为青岛发展献计策”等活动，广泛听取市民建议。

　　全面推进“创建高绩效机关、做人民满意公务员”工作，实施了大规模公务员培训。深化行政审批制度改革，审批事项调减72%。规范财政专项资金使用管理，建立了大额临时拨付资金集体研究确定制度。严格政府投资预算管理，实行财务决算委托审查制和项目稽查制。成立奥帆赛监督委员会，对奥帆赛工程建设和资金使用进行全过程监督。实施了审计结果公告制度。加快网上审批和电子监察系统建设。切实纠正损害群众利益的不正之风，治理商业贿赂等专项工作取得成效。

　　成功举办2008北京奥运会，是中华民族的百年企盼；成功举办一届“有特色、高水平”的奥帆赛，是全市人民的共同愿望。五年来，我们认真履行申奥承诺，践行“绿色奥运、科技奥运、人文奥运”理念，全面落实奥运行动规划，成功举办2006、2007青岛国际帆船赛，基地建设、城市运行、赛事组织、安全保卫、志愿者服务等顺利通过奥运测试。五年来，奥运效应持续显现，城乡基础设施加快建设，城市生态环境日益改善，城市文明和市民素质明显提升，大众健身活动蓬勃开展，全民动员工作机制基本形成，为办好2008奥帆赛奠定了坚实基础。

　　各位代表！回顾五年，刚刚过去的2007年，是青岛发展史上特殊而重要的一年。在中共青岛市委的坚强领导下，全市人民站在高起点，瞄准大目标，实现新跨越，揭开了青岛发展的崭新一页。预计全市生产总值突破3700亿元，比上年增长16%；地方财政一般预算收入达到292.6亿元，增长29.5%；城市居民人均可支配收入和农民人均纯收入增长16.5%、14%。

　　一年来，结构调整取得新进展。年初确定的武船重工等89个重点项目全部开工。船用大型柴油机曲轴竣工投产，1000万吨炼油投料试车，国内首列时速300公里动车组成功下线，一汽轻型车公司及汽车研究所落户青岛。规模以上工业增加值达到1700亿元，增长22%。服务业增加值、社会消费品零售总额分别增长17%、18.8%，均创新世纪以来最高增幅。服务业占全市生产总值的比重达到43%。港口货物、集装箱吞吐量达到2.65亿吨和941万标箱。空港客流量达到787万人次。旅游业总收入和接待国内外游客分别突破400亿元、3000万人次。农业生产形势良好，县域经济实力增强，制定出台《关于推进县域经济又好又快发展的若干意见》，县域生产总值增幅高于全市2个百分点。开放型经济持续发展，外贸进出口总额超过430亿美元，增长18%；实际到账外资24亿美元，增长31.8%，成功举办亚欧会议中小企业贸易投资博览会。

　　一年来，发展方式转变迈出新步伐。3家企业实验室进入国家重点实验室行列，中科院青岛生物能源与过程研究所奠基，国家海洋科学综合考察船项目落户我市。实施了智能交通、无线射频等技术创新项目。节能减排力度加大，中日循环型城市合作、小涧西生活垃圾处理发电等项目进展顺利。节约集约用地进一步加强，建立了土地利用评价指标体系，出台了加强地下空间土地使用权管理办法。省级以上园区投资强度和单位产出效益分别提高20%、15%。

　　一年来，城乡规划建设管理再上新水平。胶州湾北部高新区开发建设开始启动。恢复了城市规划委员会和专家咨询委员会制度，完善了规划审批行政责任追究制。海底隧道、海湾大桥等重大基础设施项目进展顺利，即平高速主线、青莱高速和荣乌高速支线青岛段建成通车，高速公路通车里程超过700公里。我市被国家规划为全国综合交通枢纽。城市管理年活动全面开展，市容环境保清洁、道路交通促畅通、景观亮化出精品收效明显。

　　一年来，民生保障实现新突破。年初确定重点办好的10件实事全面完成。住房建设“2221”目标顺利实施，18个“两改”项目全部开工，拆迁户数和面积超过前三年的总和；市财政保障性住房支出5.8亿元，超过2001年建立廉租住房制度以来投入总和。400户孤老、残疾低保家庭喜迁新居。住房价格涨幅低于全国大中城市平均水平。实现城乡就业31.3万人，城镇登记失业率2.73%。城乡中小学校和托幼园所全部实施校方责任险，农村中小学现代远程教育工程进展顺利。市区各类人员全部纳入基本医疗保障体系，社区药品“零差率”全面启动。市区新增集中供热面积360万平方米，提高了供热温度标准，建立了试供热制度。对政府特许经营公用企业，试行以市民满意为标准的考核评价制度，服务质量进一步提高。

　　一年来，文明平安创建取得新成果。大力实施人文奥运，全面启动“千帆竞发2008”工程，广泛开展文明和谐创建活动。举办了国际音乐大师班、首届全国小提琴考级优秀选手展演赛、首届中国国产电影交易会等活动。三部作品入选“五个一工程”奖。在第六届全国城运会上，青岛代表团获得第六名的好成绩。实现全国双拥模范城“六连冠”。加强基层民主建设，创新社会管理体制。做好矛盾纠纷排查化解，社会治安综合治理进一步加强。创建全国食品最安全城市，产品质量和食品安全专项整治初见成效。道路交通安全专项整治、企业安全生产监管力度加大。

　　各位代表！五年来青岛经济社会发展的大好局面，来之不易，需要倍加珍惜。五年来的生动实践，印证了中央、省市委的科学决策和战略部署，记载着全市人民的团结奋进和创新拼搏，凝聚着社会各界的聪明智慧和辛勤汗水。五年来的巨大变化，得益于历届市领导班子打下的良好基础，得益于各民主党派、人民团体和各界人士的大力支持，得益于驻青部队和武警官兵的无私奉献、驻青单位的积极配合、海内外朋友的关心帮助。在此，我谨代表市第十三届人民政府，向所有参与、支持、关心青岛发展的同志们、朋友们表示衷心的感谢和崇高的敬意！

　　我们也清醒地看到，本届政府工作与全市人民的期待相比还有差距和不足：一些工作人员公仆意识、责任意识和服务意识不强，不作为、不尽责的问题依然存在；政府部门公共服务和社会管理能力需要进一步提高；个别领域的消极腐败现象时有发生，极少数领导干部的腐败行为影响恶劣。同时，经济社会发展还面临不少矛盾和困难：节能减排难度加大；服务业比重偏低；自主创新能力不强，高技能人才缺乏；国有资产监管措施需要进一步强化；城乡基础设施欠账较多，特别是农业基础比较薄弱；解决收入、住房、医疗、治安等关系群众切身利益的问题仍需加大力度，特别是食品类价格上涨较快，加重了部分低收入家庭的生活困难；奥帆筹备服务水平和赛事运行组织能力有待进一步提高；城市软实力不够强，文化建设需要加大力度。对这些问题，需要引起高度重视，采取有力措施，认真加以解决。

　　二、今后五年的目标任务和2008年工作建议

　　各位代表！中国共产党第十七次全国代表大会高举中国特色社会主义伟大旗帜,描绘了在新的时代条件下继续全面建设小康社会、加快推进社会主义现代化的宏伟蓝图；中共山东省第九次代表大会要求坚持科学发展、和谐发展、率先发展，努力实现富民强省的新跨越；中共青岛市第十次代表大会提出了建设富强文明和谐现代化国际城市的战略目标。面对新形势新要求，对今后五年和今年的政府工作提出以下建议：

　　今后五年，政府工作的总体要求是：高举中国特色社会主义伟大旗帜，坚持以邓小平理论和“三个代表”重要思想为指导，深入贯彻落实科学发展观，落实党的十七大精神和省、市党代会部署，落实国家宏观调控政策，加快工作指导转变，着力优化经济结构和转变经济发展方式，积极推进改革开放和自主创新，切实加强节能减排和生态环境保护，更加重视民生改善和社会事业进步，推动经济社会又好又快发展，努力建设富强文明和谐的现代化国际城市。

　　五年的主要目标是：

　　——在优化结构、提高效益、降低消耗、保护环境的基础上，全市生产总值年均增长13%，地方财政一般预算收入年均增长17%。提前实现人均生产总值比2000年翻两番。服务业增加值占生产总值比重每年增加1个百分点。

　　——万元生产总值能耗下降18%左右。二氧化硫和化学需氧量排放量分别下降20%和15%。建成区绿化覆盖率达到45.5%。林木覆盖率达到36%。

　　——城市居民人均可支配收入和农民人均纯收入年均分别增长10%。实现城乡就业100万人，城镇登记失业率控制在4%以内。农村适龄人员养老保险参保率达到85%。市区集中供热率达到70%以上。

　　——高等教育毛入学率达到40%,高中阶段入学率达到95%。全社会研发投入占生产总值比重达到2.8%。文化产业增加值占生产总值比重达到6.5%。人均期望寿命达到78.87岁，人口自然增长率控制在5％。以内。

　　实现上述要求和目标，需加快推进三大任务：

　　（一）加快结构调整和发展方式转变，促进城乡协调发展，努力建设富强青岛。围绕市十次党代会提出的“三个基地、三个中心”建设目标，按照走中国特色新型工业化道路的要求，着力培育现代产业体系。大力发展制造业集群，力争“十一五”末，年营业收入过1000亿元的特大企业2家，过100亿元的企业15家，过10亿元的企业100家，五年内建成全国重要的船舶和海洋工程基地、高速列车产业化基地、汽车制造出口基地和世界知名的家电电子产品生产基地。围绕加快形成以服务经济为主的产业结构，规划建设30个服务业园区，推进300个投资过亿元项目，扶持100个骨干企业，培育100个服务名牌。围绕社会主义新农村建设，推进城乡一体化发展，基本完成市区工业企业转移任务，到“十一五”末，县域生产总值、地方财政收入占全市比重分别达到70%和40%。围绕创新型城市建设，完成“十大领域关键技术攻关”，建成“十大高新技术产业化基地”，打造“十大创新团队”。深化体制机制改革，加快国有资本从竞争性领域退出步伐，国有经济分布行业从71个减少到20个左右。整合政府投资资源，进一步提高投融资能力。完善开放型经济体系，扩大对外交往，形成参与国际经济合作竞争的新优势。围绕生态市建设，全面推进节能减排，加快发展循环经济，切实加强环境保护，走生态文明发展道路。海底隧道、跨海大桥建成通车。实施“依托主城、拥湾发展、组团布局、轴向辐射”的城市空间战略，构筑“一主三辅多组团”的现代化城市框架，打造环胶州湾地区核心圈层，在全省“一体两翼”和海洋经济战略格局中发挥更大作用。

　　（二）加快实施文化强市战略，增强城市软实力，努力建设文明青岛。坚持社会主义先进文化前进方向，以社会主义核心价值体系引领城市文明，用中国特色社会主义共同理想凝聚力量，大力弘扬民族精神和时代精神，广泛开展精神文明创建活动，形成“知荣辱、树新风、促和谐”的社会新风尚。推进文化创新，以大众文化活动为抓手，以项目建设为载体，培育新的文化业态，加快完善覆盖城乡的公共文化体系，促进文化发展繁荣。扶持公益性文化事业，发展社区文化、企业文化、村镇文化、校园文化，推进文化资源共享，推出一批有青岛特色的文艺精品。建成青岛大剧院等一批重点文化设施。促进文化产业发展，培育文化产业主体，形成一批有较强竞争力的文化企业集团。深化“诚信青岛”建设，完善社会征信体系。繁荣发展哲学社会科学，推动优秀成果和优秀人才不断涌现。

　　（三）加快发展社会事业，着力解决民生问题，努力建设和谐青岛。以“五有”为目标，提供均等化的公共服务，完善普惠性的社会保障，营造公平性的发展机会，不断开创共建共享和谐社会新局面。优先发展教育，重视学前教育，促进义务教育均衡发展和高中教育普及，推动职业教育、高等教育提高水平，实现学有所教、公平发展。支持自主创业、自谋职业，切实维护劳动者合法权益，全面落实企业工资支付规定和工资指导线制度，连续三年提高企业退休人员养老金水平，促进家庭财产普遍增加，实现劳有所得、共同富裕。推进健康城市建设，健全城乡基本医疗卫生制度，“十一五”末建成国医堂、市妇幼医疗保健中心、北部医疗中心和西海岸医疗中心，实现病有所医、健康生活。逐步提高最低生活保障标准和社会救助水平，完成老龄事业发展“1234”目标，实现老有所养、困有所济。完善多层次住房保障体系，“十一五”末基本解决市区低收入家庭住房困难，五年内完成市区旧城区和城中村改造，实现住有所居、安居乐业。增强城市公共安全保障能力，深入开展平安创建活动，确保社会安全发展、人民幸福安康。

　　各位代表！今年是全面贯彻落实党的十七大精神和省、市党代会部署的重要一年，是实施“十一五”规划承上启下的一年，也是承办奥帆赛的决战之年。政府工作要坚持“稳中求进、好字优先、改革开放、以人为本”的指导原则，全面落实国家宏观调控政策，充分认识经济运行环境的复杂性、多变性，妥善应对可能遇到的困难和风险，抓住机遇、积极进取，努力做好各项工作，努力化解突出矛盾和问题，努力保持平稳较快发展的良好态势。

　　经济社会发展的主要目标是：全市生产总值、地方财政一般预算收入分别增长13%和17%；城市居民人均可支配收入、农民人均纯收入均增长10%；城镇登记失业率控制在4%以内；万元生产总值能耗下降4.5%，二氧化硫和化学需氧量排放量分别下降7%、4.5%；人口自然增长率控制在6％。以内。

　　重点做好以下十项工作：

　　（一）加快提高城市创新能力

　　全面推进青岛高新区建设。以高端化、集约化、生态化为目标，积极探索创新发展模式，努力把高新区建成全市体制机制创新的先行区、高新产业的集聚区、生态文明的示范区。高起点编制发展规划，超前筹划基础设施建设。发挥核心区的承载和引领功能，加快引资引智步伐，增强自主创新技术孵化能力，壮大高新技术产业群，培育特色产业园。积极推动青岛崂山科技城、即墨软件园建设发展。

　　完善技术创新体系。强化企业主体地位，培育创新型企业，逐步形成技术创新梯队优势。修订《青岛市认定企业技术中心管理办法》和评价指标体系，加强对企业技术中心建设的规范引导。继续探索产学研一体化新机制。依托国家重点实验室、工程中心、研究所等各类研发机构，加快形成与重点产业发展配套的研发支撑体系。完成中科院青岛生物能源与过程研究所建设，开工建设青岛海洋科学与技术国家实验室、中海油重质油加工工程技术中心等。加快新材料等高新技术产业化基地建设。争取大学科技园成为国家级孵化器。

　　推进关键技术攻关突破。加强家电电子、生物技术、海洋科技等技术攻关，引进光伏电、集成电路、智能玻璃等一批高新技术项目。加强食品药品安全和重大疾病防治等社会公益性技术研发应用。开展技术预见研究。

　　进一步优化创新环境。推进“222”引才工程。着力培育生物技术等创新团队。建成东海岸教育实训基地，规划建设现代物流等四个公共实训基地。健全高层次创新人才评价、选拔任用和激励保障机制。政府科技投入更多地支持创新成果转化。加快建设市区两级科技创新服务平台。实施标准化战略，推动优势技术成为国家、行业和国际标准。出台知识产权战略纲要，加强知识产权保护。

　　（二）大力发展先进制造业

　　加快制造业结构调整。坚持产业集群方向和园区集约导向，做强主导产业，做大新兴产业，做优传统产业。从今年起，每年滚动推进100个投资过亿元产业项目，培育壮大汽车、造船、家电、石化等主导产业；着力发展装备制造、电子通讯、环保等新兴产业；优化提高钢铁、橡胶、纺织服装、食品饮料等传统产业。今年，确保上汽通用五菱30万辆乘用车、北船重工造船坞等建成投产。积极引进和扶持产业链配套项目，鼓励中小企业融入集群发展。

　　推进制造业布局调整。完善全市工业布局规划，明确重点发展区域、优化发展区域和限制发展区域的梯次格局。制定出台市区工业企业转移改造方案，实现老企业搬迁振兴与旧城改造、县域工业化同步推进。启动董家口重工业基地建设。坚持发展品牌经济与提高自主创新能力相结合，加强品牌梯队建设，保障品牌安全，努力培育一批具有核心技术、知名品牌和国际竞争力的企业集团。

　　组织好经济运行调控。着力监控年销售收入过10亿元的重点企业和重点产品。加强煤电油运综合协调，确保电煤安全库存和成品油市场稳定。强化部门与区市联动的企业服务机制，完善直通车服务体系。

　　（三）突出发展现代服务业

　　积极拓展生产性服务业。加快金融机构及业务中心引进步伐，推动市商业银行尽快成为区域性银行，完成农村合作银行组建工作，启动村镇银行等新型农村金融机构试点，促进金融开放、集聚、创新效应进一步显现。发展以保税物流为特色的现代物流，构建以电子口岸为基础的物流公共信息平台，推进前湾港、空港、胶州湾陆路等综合物流中心和信息家电、化工橡胶等专业物流基地建设。以服务外包、软件设计为重点，加快发展科技与信息服务业，引进和培育一批具有国际影响的服务外包骨干企业，争创国家服务外包基地城市和服务外包人才培训基地。大力发展鉴证类等中介服务业。组建大型专业会展企业，办好中国国际消费电子博览会、APEC中小企业技贸博览会，积极申办2009“夏季达沃斯”年会。

　　丰富发展消费性服务业。突出发展度假旅游，加快推进港中旅青岛海泉湾、唐岛湾游艇、宝龙城市广场、啤酒城改造等49个总投资400亿元的旅游项目，规划建设崂山风景区旅游服务中心，提高旅游管理服务水平，努力建设中国最佳旅游目的地。规范房地产市场秩序，引导房地产业持续健康发展。加快现代流通服务网络建设，大力发展品牌便民连锁经营。

　　改善服务业发展环境。进一步清理和取消限制服务业发展的各种不合理规定，鼓励支持各类社会资本进入服务业领域。拓宽服务消费领域，改善消费环境，努力扩大消费需求。制定服务业发展指标体系，创新管理体制，加大考核力度。落实好家电下乡补贴政策。

　　（四）着力加强节能减排、环境保护和集约用地

　　今年，是完成节能减排五年目标的关键一年。要针对我市节能减排的形势和面临的艰巨任务，采取非常措施、非常手段，打好攻坚战。

　　大力推进节能工作。严格落实重点用能单位目标责任，逐步强制淘汰明显高于同行业能耗标准的企业和产品。继续开展工业锅炉改造和余热余压利用，关停小火电机组6.8万千瓦。加快发展海水源热泵、风力发电、太阳能等清洁能源。继续抓好建筑节能改造。安排专项资金支持企业节能技术推广应用。

　　加大环境保护和治污减排力度。加强水污染治理，完善管网配套，完成楼山河污水处理厂、李村河污水处理厂二期工程，扩建改造海泊河污水处理厂。加强崂山水库上游排污设施建设。推进大气污染防治，加快燃煤锅炉脱硫改造，强化扬尘污染和机动车尾气治理。加强工业固体废物污染防治、生活垃圾无害化处理和综合利用，开工建设垃圾焚烧发电项目，建成小涧西生化处理厂。实施治污减排动态管理，对100家重点排污企业实行总量和浓度双重控制，加大企业违法排污查处力度。建立节能减排“双控机制”，落实节能减排统计、监测、考核实施方案，对未达标的区市实施“区域限批”。积极推进中日循环型城市合作项目。切实加强胶州湾、崂山风景区的生态环境保护。进一步增强全社会的节约意识，政府部门要发挥带头作用，推动节能减排深入开展。

　　提高土地节约集约利用水平。健全依法依规用地长效机制，严格控制建设用地供应的规模、结构、布局和时序，从严利用建设用地计划指标。完善经营性和工业用地招拍挂制度。切实加强和规范新开工项目管理。强化对土地投资强度和投入产出率等指标的考核。

　　（五）扎实推进社会主义新农村建设

　　继续把“三农”工作作为重中之重，全面落实各项强农惠农政策，加快形成农民持续增收的长效机制，加快农村生产生活方式向现代转变。今年，市级财政拟安排“三农”资金21.9亿元，比上年增长51.7%。

　　大力发展县域经济。落实促进县域经济发展的政策措施，优化产业布局，培育特色经济板块,支持平度、莱西设立省级工业园区。加强现代农业建设，提高农业综合生产能力。稳定粮食生产，确保粮食安全。建成100万亩优质蔬菜示范区，积极发展现代林业、现代畜牧业和现代渔业。推进农业产业化经营，培育壮大一批带动力强的龙头加工企业，加快重点产地批发市场规范化建设。按照依法自愿有偿原则，健全土地承包经营权流转机制，鼓励引导土地适度规模经营。落实支持生猪、奶业、油料发展的各项政策，切实抓好“菜篮子”产品生产。

　　今年，为改善农民的生产生活条件，重点做好12项工作：⑴培育100个“四五”标准示范村，扶持1000个中等收入以下村庄实现“五化”目标。⑵重点支持30家农村合作组织，推进农民专业合作社、土地股份合作社、农村集体资产股份合作社建设。⑶健全基层农业技术推广体系，因地制宜设立公益性农技推广中心站，促进农民专业技术协会发展。⑷扶持100个“一村一品”示范村、10个“一镇一业”示范镇。⑸对五市农民创业基金给予补助，扶持1万名农民自主创业。⑹新改建农村公路800公里，2009年基本实现村村通柏油路。⑺完成100座小型病险水库除险加固工程。⑻新建户用沼气2万户。⑼为农村义务教育阶段学生免费提供教科书，完成农村中小学现代远程教育工程。⑽新农合筹资标准提高到每人每年100元，提高住院费用补偿标准，完善农村医疗保险运作模式。⑾改扩建村文化活动室1200处，新建“农家书屋”1000个。⑿继续扩大新型农村养老保险制度实施范围，五年内扩展到所有村庄。

　　（六）进一步深化体制改革和对外开放

　　今年，是我国改革开放30周年。要以更大的决心和力度深化改革，勇于攻坚克难，务求在重点领域和关键环节取得实质性进展。

　　积极推进国有企业、投资体制、财政体制和社会事业改革。加快国有大企业股份制改革重组，推进股权多元化，逐步解决一股独大、机制不活的问题。推动委派外部董事进入董事会试点，规范法人治理结构，健全现代企业制度。加强国有资产监管，搭建出资人财务监管平台，组建国有资产稽查机构，规范国有企业改革和国有资产产权交易行为，确保国有资产安全。推行国有资本经营预算制度。搭建重大基础设施、城乡建设、国有资本结构调整优化三大政府投资平台。加快企业上市步伐，扩大直接融资比重。进一步完善财政体制，坚持财力与事权相统一、利益与责任相对应，增加对区市财力的支持，提高区市财政的公共保障能力。积极培植税源，加强税收征管。稳妥推进事业单位岗位设置管理实施工作。深化文化体制改革，推动文艺院团形成整体合力。按照国家统一部署，做好医药卫生体制改革工作。

　　构筑开放型经济新格局。适应更加复杂多变的国际环境，树立大开放意识，把对外开放与优化经济结构、增强综合功能、提升城市形象紧密结合，积极开展多领域交流合作。认真落实推动对外开放又好又快发展的意见，修订《外商投资产业指导目录》，统一吸引外资政策。充分发挥企业主体作用和经济园区载体功能，着力提高先进制造业、现代服务业、现代农业和节能环保产业利用外资比重。加快转变外贸增长方式，实施科技兴贸和品牌兴贸战略，提高自主品牌、自主知识产权产品和高新技术、节能环保产品出口比重，大力发展服务贸易，增加先进技术设备、能源原材料和关键零部件进口。积极申请设立青岛前湾保税港区，规划建设自由贸易港区。支持有条件的企业“走出去”，开展国际化经营。全面优化开放环境，充分发挥重大项目联席会议作用，深化大通关建设，进一步改善外国人在青居住、教育、医疗条件。服务国家外交大局，扩大友好城市合作，拓展对外交往领域。加强新时期对台工作，提高侨务工作水平。继续加大国内经济合作和对口支援工作力度。

　　做大做强民营经济。加快中小企业信用担保体系建设。继续推进“五百工程”和“中小企业成长工程”，大力实施创新型民营企业发展计划，培育一批竞争力强的民营企业集团。建成一批中小企业创业基地和产业基地，抓好纺织服装等特色产业，扶持建设10个区域特色基地。

　　（七）全面加强城市规划建设管理

　　提高科学规划水平。强化城市发展战略、功能布局和行政区划研究。认真实施《城乡规划法》，充分发挥城市规划委员会作用，切实维护规划的权威性，加强执行监督。抓紧修编新一轮城市总体规划，调整环胶州湾建设发展总体规划及控制性详细规划，精心编制环胶州湾城市设计。做好土地利用、道路交通、生态环境、地下空间等专项规划编制和城区供热、供气、给排水等专业规划的修编。

　　推进基础设施建设。完成火车站、长途汽车站、快速路三期跨铁路桥改造，加快海底隧道、跨海大桥两端接线工程。开展重庆路高架快速路、308国道快速公交系统、铁路新客站、机场第二跑道等项目前期工作，力争华电青岛发电三期开工。加大北部城区基础设施建设力度。制定实施市区河道综合治理方案。规划建设太平山中央公园和百果山世博园，加快浮山生态恢复保护工程，新增绿地186万平方米。启动创办2011中国（青岛）世界园林花卉博览会的准备工作。争创首批国家生态园林城市。

　　加强精细化管理。全面落实属地管理责任，加强铁路两侧、火车站周边等重点区域综合整治，强化占路经营、违法建筑等顽症治理。规范公共信息图形标志。加强城市管理信息化建设，逐步建成数字城市管理系统，实现奥帆中心等重点区域数字化监管。

　　（八）认真解决关系人民群众切身利益问题

　　市十次党代会在改善民生方面提出了明确的目标任务，这是对全市人民的庄严承诺，要按照这一要求，真诚倾听群众呼声，真实反映群众愿望，真情关心群众疾苦，切实改善民生质量，努力让人民群众分享到更多的发展成果，感受到更多的公平正义，体验到更多的和谐幸福。

　　今年，为改善城市居民生活，重点做好10项工作：⑴市财政安排6亿元，主要用于保障性住房建设和改善旧住宅区居住条件。建设廉租住房3000套，改造24片旧城区、城中村，整治改造120栋筒子楼、4片旧住宅区。⑵调整最低工资标准和最低生活保障标准，提高企业退休人员养老金水平。加强企业工资分配调控指导，完善职工工资正常增长机制。落实国家职工带薪年休假制度。⑶安排促进就业资金3.3亿元，实现城乡就业20万人。⑷加强市场物价监管，保障重要消费品市场供应，建立低保家庭生活补贴与物价上涨的联动机制。⑸落实经济困难家庭学生资助政策，改造市区公办中小学取暖设施。⑹调整困难居民医疗救助范围和标准，在社区全面推行常用药品“零差率”。⑺建成市老年人体育活动中心，启动市级机构养老服务示范基地和基层老年活动中心改扩建工程。加大对社会养老机构的政策支持力度。⑻新增7条公交线路，更新公交车800辆，基本实现公交车进场停放。⑼新增居民集中供热面积380万平方米，普及率达到48%。⑽5月1日起，中山公园、植物园、博物馆、图书馆等免费开放。

　　切实加强“平安青岛”建设。出台创建全国食品最安全城市实施意见。深入推进食品药品安全工作，确保人民群众饮食用药安全。建立食品安全电子信息备案追溯系统和食品检验监测体系，支持两级质检中心和市区三大批发市场检测室升级改造。加强重大动物疫病防控体系建设，实现全市安全无疫情。强化安全生产监管，加强隐患排查治理和交通运输等重点领域、重点行业专项整治，实现交通运输安全状况明显好转，坚决遏制重特大事故发生。启动黄岛石化区居民搬迁工作。健全城乡公共安全应急管理体系，强化防灾减灾，提高应对突发事件能力。畅通社情民意诉求渠道，做好人民群众来信来访工作，完善“大调解”工作机制，积极预防和妥善化解各类不稳定因素。加强社会治安综合治理，推进群防群治队伍建设，改革和加强城乡社区警务工作，健全社会治安防控体系，依法防范和打击违法犯罪活动，确保社会安定。

　　（九）大力推进文化建设和社会事业发展

　　加强社会主义核心价值体系建设。用社会主义荣辱观引领社会风尚，推进社会公德、职业道德、家庭美德和个人品德建设，营造尊老爱幼、扶贫济困、礼让宽容的和谐人际关系。大力实施人文奥运工程，深入开展“文明赢得尊重”活动。强化法制宣传教育，深入实施《全民科学素质行动计划纲要》，营造遵纪守法、积极健康的舆论氛围。

　　大力推动文化繁荣发展。要把发展公益性文化事业作为保障群众基本文化权益的主要途径，加大政府投入力度。加快青岛大剧院建设，做好市科技馆、提琴博物馆、数字图书馆、图书大厦和出版物交易中心建设的前期工作。设立文化艺术政府奖项，繁荣文艺创作，活跃基层文化生活。打造“蔚蓝青岛”大型歌舞品牌，办好第二届中国国际小提琴比赛、青岛音乐节和图书文化节。大力培育文化产业，加快国家文化产业示范基地、国家动漫创意产业基地和影视传媒基地建设。扶持文化演艺、动漫游戏、新闻出版、工艺设计、影视传媒等文化创意产业发展。加强文化市场和网络监管，保障文化安全。

　　促进社会事业全面进步。坚持优先发展教育。提高义务教育经费保障水平。建立市区教师农村支教补贴制度。深入实施教育关爱工程，提高特殊教育学校学生资助标准。加强新建小区、旧城改造中的中小学校配套建设。以促进就业为导向，优化职业教育专业设置。支持驻青高校重点学科建设，加强与驻青高校的合作共建。健全医疗服务体系和公共卫生体系，完善社区卫生服务，逐步实现社区首诊、分区医疗和双向转诊。强化公共卫生监督和医疗卫生监管。规范公立医院管理制度，树立良好医德医风，提高医疗服务质量。深入实施“奥运争光计划”、“千帆竞发2008”工程。基本完成第十一届全运会青岛赛区的筹备，确保市体育馆、游泳跳水馆、综合训练馆按期交付使用，开工建设全民健身中心。做好全国第二次经济普查工作。加强国防动员和民兵预备役建设，推进军民双拥共建，加大为部队办实事力度。统筹解决人口问题，稳定低生育水平，提高出生人口素质。切实做好民族宗教工作。促进档案、史志、气象、地震、人防、仲裁、红十字、残疾人等工作不断发展。

　　（十）全力以赴办好2008奥帆赛和残奥帆赛

　　各位代表！再过216天，我们将迎来世人瞩目的奥帆赛。今年，国际国内的目光将会更多地投向青岛，这是展示青岛改革开放发展新成就、城市文明新形象，加强国际交流合作的重要机遇。一定要举全市之力，集各方之智，努力交出一份精彩完美的合格答卷。

　　高质量完成奥帆中心建设和环境整治。3月底前，奥运分村及接待中心、注册中心试运行。对奥帆中心、前海一线进行绿化、亮化、美化，加强重点区域陆源排污监管和水域环境质量监测，确保赛区环境质量达标。

　　高水平组织好奥帆赛和残奥帆赛。按照“隆重、热烈、简洁、欢快”的原则，策划组织好奥运火炬传递和奥帆赛开闭幕仪式。认真做好接待保障、志愿者服务，组织好城市运行方案、应急预案的衔接演练，构建严密的安保体系。加快推进公共场所无障碍建设，成功举办残疾人奥运帆船赛，实现“两个奥运、同样精彩”。

　　做好奥帆中心赛后利用工作。进一步放大“帆船之都”品牌效应，使奥帆中心成为国际知名的水上运动中心，成为国际体育文化交流的重要载体，成为市民游客休闲健身娱乐的重要场所，启动奥运博物馆建设，为青岛留下宝贵的奥运文化遗产。

　　我们坚信，通过全市人民的共同努力，我们一定能够不负众望，圆满实现“有特色、高水平”的目标，为青岛增添光彩，为祖国赢得荣耀！

　　三、加快建设服务型政府

　　全面贯彻落实党的十七大精神和省、市党代会部署，全面完成市十四届人大确定的各项任务，需要建设一个有执行力、公信力、创新力的政府，需要建设一支为民、务实、清廉的公务员队伍，努力塑造法治、责任、廉洁、高效的服务型政府形象。

　　（一）深入学习贯彻中国特色社会主义理论体系，提高公务员队伍整体素质

　　认真学习贯彻落实党的十七大精神，学习马克思列宁主义、毛泽东思想、邓小平理论和“三个代表”重要思想，深入开展学习实践科学发展观活动，努力用马克思主义中国化最新成果武装头脑、指导实践、推动工作。大力弘扬八个方面的良好风气，切实加强思想作风、学风、工作作风、领导作风和干部生活作风建设。继续贯彻实施《公务员法》，深入开展“创建高绩效机关、做人民满意公务员”工作，切实增强公仆意识、责任意识、服务意识，提高政府的执行力和公信力。加强经济、科技、法律、社会管理等方面的培训学习，切实提高政府工作人员依法行政、公共服务的能力和水平。

　　（二）加快职能转变，创新政府运行机制

　　深化行政管理体制改革。开展对政府运行机制和职能定位的前期研究，精简和规范各类议事协调机构及其办事机构，为新一轮行政管理体制改革做好准备。在加强和改善经济调节、市场监管的同时，完善社会管理和公共服务体系。进一步推动行业协会、中介组织的改革和发展，充分发挥行业协会、商会的作用。

　　提高科学民主决策水平。对城市规划、基础设施建设等重大事项，按照程序进行专家咨询论证。对关系人民群众切身利益的事项，实行社会公示和听证。实施重大决策法律论证和合法性审查制度。贯彻落实《政府信息公开条例》，扩大政务公开的范围和层次，多渠道、多形式开展好为青岛发展献计策活动，保障人民群众的知情权、参与权、表达权和监督权。

　　全面实施行政问责制。建立行政机关责任检讨制度。今后，因工作失误造成严重影响和后果的，有关负责人要向市民公开道歉，公布整改方案，限期改正。对行政不作为、乱作为和推诿拖延、越权办事等行为，要严肃查处。

　　（三）推进依法行政，提高政府法治水平

　　带头维护宪法和法律的权威，为全社会作出表率。自觉接受人大及其常委会的监督，认真执行人大及其常委会的决议、决定。自觉接受人民政协的民主监督，坚持政府重大决策向人大政协报告、通报。加强与各民主党派、工商联、无党派人士和人民团体的沟通联系，切实落实特约人员工作制度，认真听取意见建议。高度重视社会公众监督和新闻舆论监督，继续办好《行风在线》节目。

　　改革创新行政审批制度。完善审批事项经常性审查机制。健全网上审批和电子监察系统，基本实现审批、服务事项网上办理和监督。建成市行政服务大厅，实现80%以上行政审批和服务事项集中办理，30%现场办结。

　　加强政府立法和行政执法工作。加快节能减排、环境保护、保障民生和社会管理等方面的立法。坚持依法行政,进一步推进政府行为的法治化、程序化、规范化。完善行政执法评议考核制度，严格落实行政执法责任制。实施重点镇和中心镇相对集中行政处罚权试点。

　　（四）坚持从严治政，塑造风清气正的政务环境

　　切实加强以完善惩治和预防腐败体系为重点的反腐倡廉建设，在坚决惩治腐败的同时，更加注重治本，更加注重预防，更加注重制度建设。严格落实“一岗双责”制度。加强对领导干部特别是主要领导干部、人财物管理使用、关键岗位的监督，加强对重点专项资金、重大投资项目、重点部门的审计和监察。健全经济责任审计等制度，扩大审计结果公开范围。倡导勤俭节约，反对奢侈浪费，降低行政成本。加强行政资产管理，严格控制行政机关办公楼等楼堂馆所建设。继续深入治理商业贿赂，坚决纠正食品药品安全、环境保护、土地征收征用等方面损害群众利益的不正之风。

　　各位代表！新的一年已经开始，我们的未来充满希望，我们的事业任重道远。让我们更加紧密地团结在以胡锦涛同志为总书记的党中央周围，高举中国特色社会主义伟大旗帜，在中共青岛市委的坚强领导下，万众一心，开拓进取，为建设富强文明和谐的新青岛，创造更加幸福美好的新生活而努力奋斗！
